# Health Humanities curriculum and evaluation in health professions education: a scoping review

**DOI:** 10.1186/s12909-021-03002-1

**Published:** 2021-11-10

**Authors:** Sandra E. Carr, Farah Noya, Brid Phillips, Anna Harris, Karen Scott, Claire Hooker, Nahal Mavaddat, Mary Ani-Amponsah, Daniel M. Vuillermin, Steve Reid, Pamela Brett-MacLean

**Affiliations:** 1 Health Professions Education, University of Western Australia, Perth, Australia; 2 Faculty of Arts and Social Sciences, Maastricht University, Maastricht, Netherlands; 3 Medical School, University of Sydney, Sydney, Australia; 4 Medical School, University of Western Australia, Perth, Australia; 5 College of Health Sciences, University of Ghana, Accra, Ghana; 6 Institute for Medical Humanities, Peking University, Beijing, China; 7 University of Cape Town, Cape Town, South Africa; 8 Faculty of Medicine & Dentistry, University of Alberta, Edmonton, Alberta Canada

**Keywords:** Health professions education, Medical Humanities, Health Humanities, Curriculum evaluation, Scoping review

## Abstract

**Background:**

The articulation of learning goals, processes and outcomes related to health humanities teaching currently lacks comparability of curricula and outcomes, and requires synthesis to provide a basis for developing a curriculum and evaluation framework for health humanities teaching and learning. This scoping review sought to answer how and why the health humanities are used in health professions education. It also sought to explore how health humanities curricula are evaluated and whether the programme evaluation aligns with the desired learning outcomes.

**Methods:**

A focused scoping review of qualitative and mixed-methods studies that included the influence of integrated health humanities curricula in pre-registration health professions education with programme evaluate of outcomes was completed. Studies of students not enrolled in a pre-registration course, with only ad-hoc health humanities learning experiences that were not assessed or evaluated were excluded. Four databases were searched (CINAHL), (ERIC), PubMed, and Medline.

**Results:**

The search over a 5 year period, identified 8621 publications. Title and abstract screening, followed by full-text screening, resulted in 24 articles selected for inclusion. Learning outcomes, learning activities and evaluation data were extracted from each included publication.

**Discussion:**

Reported health humanities curricula focused on developing students’ capacity for perspective, reflexivity, self- reflection and person-centred approaches to communication. However, the learning outcomes were not consistently described, identifying a limited capacity to compare health humanities curricula across programmes. A set of clearly stated generic capabilities or outcomes from learning in health humanities would be a helpful next step for benchmarking, clarification and comparison of evaluation strategy.

**Supplementary Information:**

The online version contains supplementary material available at 10.1186/s12909-021-03002-1.

## Background

The medical humanities is a rapidly evolving field that provides an interdisciplinary approach to understanding the meaning of health, illness and disease for patients in the context of the social worlds in which they live and work, to enhance empathic and effective responsiveness to their experience and needs. A broad interdisciplinary field, ‘health humanities’ as it is increasingly referred to [1], encompasses perspectives, insights and approaches from diverse arts (e.g., visual arts, performing arts, music) and humanities (history,literature, narrative, ethics and philosophy) disciplines. As stated by Shapiro p.192, the aim is to help students of the health professions “better understand and critically reflect on their professions with the intention of becoming more self-aware and humane practitioners” [2]. The term “medical humanities” is most often associated with education of medical practitioners; in contrast, the “health humanities” broadly includes health and social care professions, with the arts and humanities contributing to education, research and health care practice [3, 4].

In addition to enhancing knowledge and understanding across a variety of realms, the ‘health humanities’ are also viewed as important for developing the skills, behaviour and attitudes that  health professionals need to become clinically excellent, creative and critically reflexive practitioners. Increasing calls for humanizing medicine has seen the introduction of the medical and health humanities as an expanding global movement [5]. Health humanities offerings range from one-off co-curricular interventions, such as visits to art museums, electives, both optional and mandatory lectures and courses, to fully integrated, longitudinal curricular themes. Though there is a wealth of evidence that the arts and humanities are highly valued as an approach [2–4, 6], the knowledge base about the impact of these interventions is currently scattered and ad-hoc. The articulation of learning goals, processes and outcomes related to the introduction of the humanities into health professions curricula, requires synthesis. Curriculum designers and instructors need a generative framework for evaluating health humanities courses. Curriculum evaluation hinges on measuring whether the graduate learning outcomes of a course or programme have been met, by determining whether the desired change in the learner’s attitudes, knowledge, skills and behaviour has been achieved [7].

Despite the increasing popularity of arts and humanities-based approaches to health professions education, reviews that have explored the contributions of health humanities to desired learning outcomes in health professions education have found a paucity of evidence [6, 8–10]. In Moniz’s [11] recent large-scale overview of the rich and diverse use of arts and humanities they found that just over half of the 769 publications included in their review were evaluated; and in only 27% of the publications were learners assessed. They concluded that the published literature regarding arts and humanities contributions to medical education are characterized by brief, episodic instalments and largely lacking a theoretical lens that may support accumulation of evidence into an “overarching theory of practice”—presenting a formidable challenge to characterizing and evaluating health humanities learning and teaching [11].

Recognizing these difficulties, Dennhardt [12] conducted a scoping review and synthesis of quantitative outcome studies of medical humanities that led to the development of a conceptual framework of epistemic functions of arts-based teaching to support curriculum development and evaluation in health professions education [12]. They identified 1) three focuses, or different ways arts-based teaching are used (as expertise, dialogue, and expression/transformation) and 2) related knowledge purposes (for mastering skills; interaction, perspective-taking, relational aims; personal growth/ activism). Haidet [13] similarly developed a conceptual framework to guide careful design, contextualization, and evaluation of arts-based learning. To maximize arts-based learning outcomes, they recommend that the unique qualities and affordances of different arts-based forms be assessed and used to inform engagement, meaning-making, and knowledge translation strategies and processes when facilitating arts-based approaches to health professions education. To date, however, an evaluation framework has not been proposed for health humanities teaching and learning. This is likely due to the tensions that exist between scientific, positivist learning and humanistic, constructivist learning, and the different approaches needed to measure outcomes that are believed to be quantifiable and objective, compared with impacts that are more subjective, subtle, and continuous  [6]. As noted by Dennhardt [12], health humanities teaching cannot easily be systematised in relation to simple descriptive categories. In the context of the competence and outcome-based curriculum frameworks commonly used in the health professions, the heterogeneity of the health humanities can make it very difficult to integrate them into core curricula and may be one of the reasons why it often remains an elective offering. Additionally, the epistemological features of subjects may provide a strong prima facie justification for handling those subjects in certain ways within the curriculum [14].

Most prior reviews have focused on quantitative studies of medical/ health humanities teaching. Compared to these more reductionist approaches, the research team for this study believed that qualitative and mixed methods studies would provide a more robust understanding of why and how arts and humanities are used and evaluated in health professions education. As such, we undertook a scoping review of qualitative and mixed-methods studies of health humanities curricula in pre-registration health professions education to provide a basis for the development of a curriculum and evaluation framework for health humanities teaching and learning that would enable comparability of curriculum offerings and outcomes. As an international team of scholars and practitioners with expertise in health humanities, health professions education and health care, we were also interested in developing a framework that would be applicable across a global context.

The following questions guided our review:

1. How, and why, are the health humanities used in health professions education?
  - *What is the focus of health humanities teaching?*
  - *What domains, and levels of learning are addressed?)*
2. How are health humanities curricula evaluated?

For the purpose of this review, we considered ‘health humanities’ as being inclusive of ‘medical humanities’.

## Methods

We conducted our review in accordance with Arskey and O’Malley’s framework for scoping reviews [14]. While a scoping review provides a systematic approach to mapping literature on a given topic to provide a comprehensive picture of the literature, it does not make discriminations based on the ‘quality’ of the studies as occurs with systematic reviews [15]. This allowed for reflexivity through the process of extracting data to develop a descriptive, narrative synthesis of the selected publications, leading to clarification and refinement of guiding questions and methods as understanding of the literature becomes clearer.

### Search strategy

To identify the relevant articles for consideration, a comprehensive search strategy was applied using the Cumulative Index to Nursing and Allied Health Literature (CINAHL), Educational Resources Information Centre (ERIC), PubMed, and Medline using keywords including combinations of “student*”, “health professional*” AND “education”, “curricul*”, “programme”, “teaching”, “learning”, “evaluation”, “assessment” AND “health humanities”, “medical humanities”, “arts”. Publications between March 2015–November 2020, available in English, in peer reviewed journals were searched. The initial search was undertaken using the keywords and inclusion/exclusion criteria from April 15 to 20, 2020 identifying 8594 articles. The search was repeated on November 22, 2020 with a further 27 articles identified bringing the total number of articles included in the scoping review to 8621.

### Inclusion and exclusion criteria

Population: Of interest were  health professions students, including medicine, nursing and allied health professional students, undertaking a pre-registration programme or course of studies at a university. These could be undergraduate, or graduate-entry programmes that led to the ability to become registered health practitioners. Studies focused on participants or students who were not enrolled in a pre-registration health professions course were excluded.

Intervention: Learning interventions (activities) using health humanities integrated into curricula with a focus on the achievement of stated learning outcomes/objectives and associated curriculum evaluation were included. Studies that focused on ad-hoc health humanities learning experiences (e.g., a once off visit to an art gallery), rather than integrated course content (e.g., a seminar series developing students skills in observation) were excluded.

Outcome: Any assessment or programme evaluation of the “impact”; “outcome*”; “benefit”; AND the achievement of “attributes”; “skill*”; “knowledge”; “behaviour”; “personal growth” or “reflect*”; “transformation” were searched for; only articles meeting these criteria were included. Articles that did not report clear outcomes were excluded.

### Article screening and selection

**Fig. 1:**
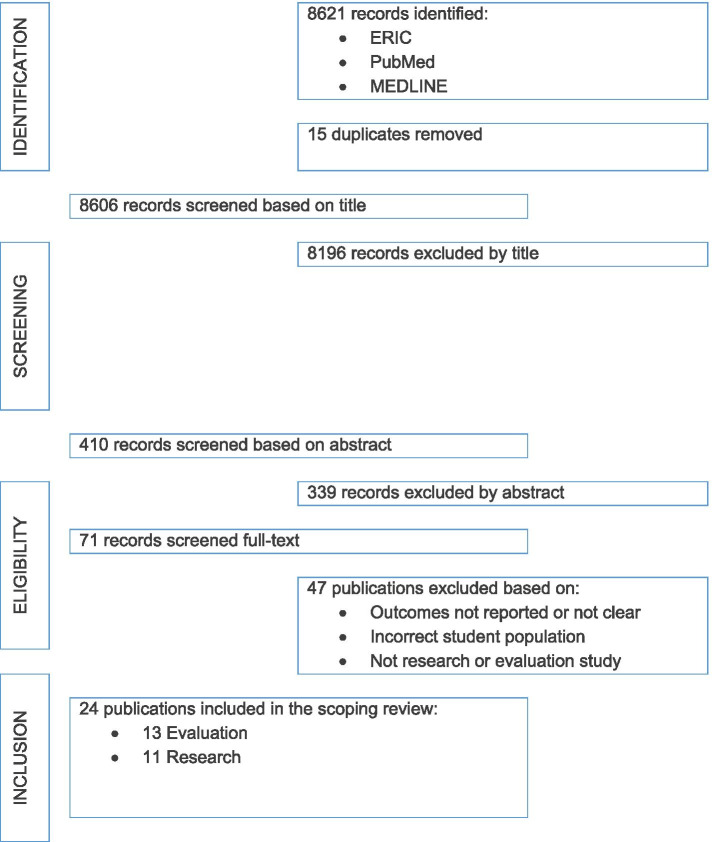
PRISMA diagram summarising study selection process

Following removal of duplicates, 8606 titles were reviewed, each by two reviewers (CD, SC, BP, FN, KS, PB, CH). It was at this stage that publications were screened to ensure that they were qualitative or mixed-methods studies. Clearly non-empirical (conceptual, theoretical contributions, as well as descriptive articles) and reviews were excluded. Subsequently, 410 abstracts were each reviewed by two members the project team (SC, BP, CH, KS, PB, FN). Additional non-empirical articles were then excluded, as well as empirical studies that only reported quantitative findings. From this, 71 papers were included for full paper review, each by two members of the project team (SC, BP, PB, KS, FN, CH, MA) and 24 papers were then identified for full data extraction. Hand searching of references for this final set was also completed, which did not identify any additional articles for inclusion (SC) (Fig. 1).

### Data charting

We developed a standardized listing of data fields to facilitate a descriptive, narrative synthesis of the data. Form fields that were used to extract data from the included articles into an Excel spreadsheet included: 1) article citation elements and 2. Health humanities curriculum intervention and programme evaluation details (see Table 1). Two reviewers extracted the data (FN, DC) that was subsequently checked by an independent second reviewer (SR, NM, KS, SC). Any conflicts were resolved by discussion (SC, FN, DC).

**Table 1:** Data extraction fields

Article citation elements	Health Humanities Curriculum Intervention and Programme Evaluation Details
Authors Title Year of Publication Journal Country of Publication Article Type (Research/ Study Design or Programme Evaluation)	Student Population Health Humanities Discipline(s) Health Humanities Learning Foci^a^ Stated Learning Outcomes/Objectives Level of Learning (Bloom)^a^ Learning Domain (Knowledge, Skills, Attitudes)^a^ Type of Educational Intervention: Delivery Mode, Duration of intervention Assessment of learning (Formative/ Summative) Level of Programme Evaluation (Kirkpatrick’s)^a^

^a^Variables used for secondary analysis

The process of data coding was iterative and led to refinements in our approach to analysing the data as our understanding of the articles included in our review evolved. The initial analysis was descriptive with basic information extracted including reference citation elements such as year of publication, country of publication and type of article which was coded as “evaluation”, i.e., focusing on programme evaluation, or “research”, i.e., focused on answering specified research questions and study design (“qualitative” or “mixed method”). In addition in this phase the type of student participants, the health humanities disciplines involved, mode and duration of learning, learning outcomes and assessments described, along with whether an educational theory or framework was specified were recorded and are summarised as frequencies in the findings.

The secondary analysis considered the impact of the learning experiences in relation to the Bloom’s domains of learning: knowledge (cognitive), skills (psychomotor), attitudes/behaviours (affective) and six levels of learning: remember, understand, apply, analyse, evaluate and create [16]. It also considered the foci of health humanities teaching as informed by previous reviews [8–10, 12, 17], as well as insights of the authors’ team who all have experience using and studying the arts and humanities in their teaching and research. Thus we identified six foci for health humanities teaching and learning:

1. knowledge acquisition.
2. mastering skills (observation, listening, reflection) [12];
3. interaction, perspective taking, and relational aims (person-centred communication, compassion, empathy) [12];
4. personal growth and activism (transformation, values, professionalism) [12];
5. personal wellness and self-care (stress management, mindfulness, resilience building) and.
6. critical evaluation (evidence synthesis) [3].

Ambiguous data were analytically discussed by research team members and final coding decisions were agreed upon by consensus of three researchers (SC, FN, DC). Synthesised results are summarised as frequencies of occurrence for the domains of learning, level of learning and health humanities foci.

The evaluation strategies applied in each included paper were also classified using Kirkpatrick’s four-level training evaluation model, encompassing: 1) process evaluation (participant satisfaction), 2) content evaluation (knowledge, skill change), 3) impact evaluation (change in behaviour), and 4) outcome (change in practice) and classified as applying both formative and summative programme evaluation [18, 19].

## Findings

Our selection strategy identified 24 articles for inclusion in this scoping review. The full details of these papers are available as the supplementary material Additional file 1: Appendix A. Most of the papers were published in 2016 (*n* = 6) and 2017 (*n* = 9); over half were published in North America (*n* = 13); the remaining authors were based in England, Ireland, Australia, India, New Zealand, Spain and Sweden. Thirteen articles were classified as evaluation studies, (focusing on programme evaluation) and 11 were coded as research studies (answering specified research questions). Fifteen articles applied mixed methods approaches to data collection and nine used qualitative methods with the prevalent analysis techniques being descriptive and thematic analysis. All the included studies reported findings that were supportive of health humanities educational activities and interventions for pre-registration health professions students and reported positive learning environments and experiences.

The educational interventions described in the article set covered a wide range of health humanities disciplines and learning activities. Interventions were mostly balanced between arts-based (visual, performing arts, and music; *n* = 10); humanities-based (reflective practice, literature/ narrative medicine; film/cinema; ethics/philosophy, *n* = 11); and multidisciplinary approaches, (*n* = 3). Most interventions were directed to medical (*n* = 12) and nursing (n = 10) students. The numbers of students reported as participating in the studies included in our data set ranged from 9 to 477 individuals. Only one intervention was delivered exclusively online [20]; the remainder involved face to face learning. Six articles did not state the length of time the intervention lasted for, three stated the activities lasted for a single session of between 2 to 6 h and the remaining 15 health humanities learning innovations lasted for between 4 weeks and a year.

### How, and why, are health humanities used in health professions education?

The health humanities educational interventions described in the final set of studies were widely varying; the one commonality they all shared was that they differed from traditional educational interventions used in the health professions in relation to both intent and form. They tended to focus on the “human side of medicine” (practitioner, patient, health care systems), and tended to use more active, transformational forms of learning, compared to more passive, informational forms (such as lectures, tutorials and laboratory sessions).

Table 2 summarizes three pertinent descriptive elements in relation to the current review: health humanities discipline(s); domains of learning addressed; and the level of learning. A broad range of arts and humanities disciplines were used. Most of the health humanities interventions aimed to address attitudes and behaviour, or the affective domain of learning (*n* = 10); the remainder addressed knowledge and skills-based domains about equally. Most of the interventions were directed towards expanding understanding (*n* = 10) and applying new learning (*n* = 7), learning levels 2 and 3.

**Table 2:** Main descriptive elements of health humanities articles included for analysis (*n* = 24)

Health Humanities intervention	Count of Articles	Article #s- refer to Appendix A
Reflective practice (includes reflective writing)	5	#6, #10, #11, #13, #23
Visual arts-based (includes art therapy)	4	#1, #5, #19, #22
Performance (drama, simulation-based learning)	4	#3, #4, #12, #15
Multidisciplinary	4	#9 #17, #18, #20
Literature/ Narrative Medicine (includes creative writing)	2	#8, #21
Film/Cinema	2	#14, #16
Music-based learning (includes music therapy)		#24
Ethics/Philosophy	1	#7
**Domains of Learning (Bloom et al. 1956)**		
1. Knowledge (Cognitive)	7	#2, #4, #7, #14, #17, #19, #20
2. Skills (Psychomotor)	7	#1, #5, #6, #9, #10, #15, #24
3. Attitudes/Behaviours (Affective)	10	#3, #8, #11, #12, #13, #16, #18, #21, #22, #23
Bloom’s Six Levels of Learning		
1. Remember	0	NIL
2. Understand	10	#2, #3, #4, #14, #16, #17, #18, #20, #21, #22
3. Apply	7	#10, #11, #13, #15, #19, #23, #24
4. Analyze	3	#7, #8, #9
5. Evaluate	2	#1, #12
6. Create	2	#5, #6

It is noteworthy that of all the data we charted, domain and level of learning proved challenging in almost half of the cases (n = 10). Some described interventions that aimed to address attitudes and behaviour, however delivered content in the cognitive (knowledge) domain [21]. In other cases, interventions directed to educating students about the value of seeing a situation from another’s perspective aimed to reach but did not quite meet the benchmark for higher learning levels beyond “understanding”. For example, Campbell [22], Centeno [23]; Gilkison [24] partially facilitated students’ exploration of attitudes and values to provide a foundation for future professional behaviours and practices, but did not extend the learning to the level of analysing, integration or creation. In some cases, studies did not report clear learning outcomes or levels of learning – in these cases, what was reported was delivered sometimes did not align.

Table 3 summarizes the foci or proposed function of the health humanities interventions included in our review. There is overlap between Bloom’s learning domains, and the first three foci are listed for health humanities curricula in this table. Most articles were coded as having multiple foci; the large majority used health humanities interventions for the purpose of developing and mastering skills (*n* = 20) to promote development of capabilities associated with patient-centred care. Interestingly, half of the studies included a focus on enhancing knowledge to support humanism (*n* = 12; this was the primary focus in four studies), which might be considered a low-level objective. Just over half (*n* = 11), included a focus on personal growth and activism (formation/ transformation). Fewer educational activities focused on using the health humanities for critical evaluation and only one article used health humanities practices for promoting well-being of the developing health professional [25].

**Table 3:** Foci of health humanities evaluation outcomes

Foci	Count of Articles	Article #‘s (refer to Appendix A)
1. Health Humanities for knowledge	13	#3, #4, #5, #7, #9, #10, #13, #14, #16, #18, #20, #22, #24
2. Health Humanities for developing and mastering skills (observation, listening, reflection)	20	#1, #2, #3, #4, #5, #6, #7, #8, #9, #10, #12, #14, #16, #17, #18, #20, #21, #22, #23, #24
3. Health Humanities for interaction and communication (person-centred, compassion, empathy, inter professional,)	20	#2, #3, #5, #6, #7, #8, #9,#10, #12, #13, #14, #15, #16, #18, #19,#20, #21, #22, #23, #24
4. Health Humanities for behaviour formation and transformation (personal growth, values and activism, professional behaviour, cultural sensitivity)	12	#2, #3, #7, #8, #9, #10, #11, #12, #20, #21, #22, #23
5. Health Humanities practices for personal wellbeing and self-care (stress management, mental health first aid, health promotion, resilience)	1	#11
6. Health Humanities for critical evaluation (evidence synthesis)	4	#5, #8, #11, #22

### How are health humanities curricula evaluated?

To begin, none of the studies referred to a specific evaluation or other theoretical framework that had been used to guide the evaluation of their health humanities curricula. Many did specifically describe their evaluation effort as either formative (*n* = 6) or summative (*n* = 15). With respect to Kirkpatrick’s Level of Evaluation, most of the studies assessed participants’ response to and satisfaction with the learning experience (Level 1); for a quarter of the studies (*n* = 6), this was the *only* evaluation that was conducted [22, 25–29]. For these studies, there was little association between the evaluation and the intended learning outcomes. Fourteen of the review studies evaluated the health humanities intervention at Level 2 [20, 23, 30–40]. These studies evaluated the capacity of the health humanities curricula to enhance a student’s knowledge, or skills, or both - linking the intervention with the intended learning outcome. Only three studies [24, 41, 42] evaluated health humanities educational interventions in relation to their impact on changing participant behaviour (Level 3). The study by Haidet [43], aimed at the highest level of evaluation and was able to demonstrate that compared to students in the control group, students in the health humanities course demonstrated statistically significant and educationally meaningful gains in adaptability and listening behaviours [13].

With respect to evaluation methods, most of the studies conducted a post-curriculum evaluation, via a survey instrument, focus group, or interview. Three studies included pre−/post-test evaluation [25, 33, 35]. Assessment of learning is often used to evaluate health humanities curricula: reflective writing and narrative essays were used to assess the value of health humanities curricula in seven studies [20, 29, 35, 39–42]. These were not used to assess higher levels of learning (such as creating new understandings) but aimed at developing and practising the skills of reflection so they could be applied to future health care practice. The students also identified in the evaluations that they had learned about themselves in each of these seven papers. The risk of bias due to missing results was minimised by having two team members agree on the data extraction, which also enhanced the confidence in the reported synthesis of results.

## Discussion

The findings of this review confirm the findings of previously published quantitative systematic reviews surrounding health and medical humanities curricula [8, 10, 11] but the inclusion of qualitative data adds further clarification and a depth of understanding of the learning outcomes or core capabilities being addressed through health humanities learning activities and how these curricula are being evaluated. The primary finding of this review was that there is an absence at present of a consistent framework for health humanities learning, teaching and assessment, and hence, little capacity for systematic evaluation within or across curricula. Many included articles did not report clear learning outcomes or levels of learning meaning that in some instances, what they intended to teach and what they delivered sometimes did not align. Other articles identified that the learning was not a linear process, which meant that the achieved learning outcomes were not always the planned learning outcomes. For example, Patterson [21] identified the heterogeneous nature of learning outcomes achieved by students engaged in a medical humanities module. While many papers made generalised statements about enhancing students’ knowledge, skills and values, specific learning outcomes were not presented in a cohesive or consistent manner that would facilitate comparisons across schools in different contexts. This made it very difficult to comment on the similarities and differences in approaches taken or in the learning that was achieved and is a limitation of this review. Combined, these factors mean there is currently a limited capacity to compare health humanities curricula across programmes. An internationally developed, empirically based, locally adaptable set of clearly stated generic capabilities or outcomes from learning in health humanities would be helpful for benchmarking, clarification and comparison.

Insofar as there was a key learning outcome, framework or focus across the studies, a second finding was that health humanities teaching focuses on developing students’ *perspectives* and hence, on developing skills in *reflexivity*. Development of perspective involves the capacity to see the complexity of situations surrounding health. For example, Gilkison [24] analysed reflective writing and discussed how the students had learned about themselves, others, and their health professional practice, through experiencing emotional responses contained within narratives. Another common learning outcome across several of the educational interventions was the development of *capacity for self-reflection* or introspection. Others reported similar evaluation findings where the students re-conceptualized their future roles as health professionals and how they would interact with patients and families in a more reflective and *person-centred* way [21, 23]. This person-centred approach focused on communication that is *empathetic* and which is reported elsewhere as one of the main aims of health humanities-based curricula [6, 12]. However, there was little published evidence that these aspirations are carried through to observable changes or outcomes later in the curriculum or post-graduation.

The studies captured in this review also indicated some of the tensions or challenges that health humanities teaching must confront. For example, a third finding was that some of the included studies aimed to address the affective domain in Bloom’s Taxonomy of Learning [16] but often delivered content in the cognitive domain. We suggest that this common situation arises in part because of the intensive resourcing required for affective learning (for example for small group or one to one teaching and guided reflection). It may also represent tensions and challenges in achieving authentic affective learning, for example, between learning offered on the basis of intrinsic value, whose qualities may be altered by the very act of assessment. These findings support the need to scaffold health humanities specific teaching vertically through the whole curriculum rather than being confined to the earlier years as is often the case. However, it is well known that this is more challenging to accomplish later, in what is typically the clinical space in curricula.

Related to this was the fourth finding that most of the evaluations focused on process and content, with only three of the studies evaluating changes in behaviour [24, 41, 42]. Interestingly, only four papers focused on developing skills in critical evaluation [20, 25, 33, 40]. So, while the focus of health humanities learning in the remaining studies, was to develop knowledge, communication and interaction skills, for personal growth and professional behaviours, the activities reported did not include critical evaluation. The critical health humanities or practise of *evidence synthesis* are seen as being very important in ensuring the capability of having perspective and is supported by evidence in health humanities [3].

### Limitations

The data charting and data extraction processes required interpretation of the findings reported in the included articles. While steps were taken to minimise any mis-interpretation or the introduction of bias during the data charting and data extraction process, this may be a limitation of the review. Other limitations of the review include the possibility that the search strategy missed publications that would have met the inclusion criteria or an article may have been excluded incorrectly.

## Conclusions

The findings of this review suggest the next step is to articulate a set of core capabilities for health humanities. The value of core capabilities for developing health humanities curriculum *within a programme* would be twofold: first, to more systematically develop integrated learning activities that can achieve some of the higher-order educational outcomes desired; and secondly, to more accurately and systematically evaluate whether these core capabilities are being achieved. If we are to see health humanities education realise changes in socialised health care practices that put the patient/person at the centre of care, they must move towards expecting students to analyse, integrate, evaluate and create or form new knowledge, new perspectives and enact new behaviours. A framework of core capabilities will enable educators to identify where current activities do not achieve these aims despite their intentions.

Comparison *across and between programmes* is an important source of innovation in education, becoming all the more important in a globally connected world. Because the health humanities are heterogeneous globally, any framework for comparison must be sufficiently flexible as to allow for localised priorities, cultural needs, and learning traditions and practices.

Finally, this review revealed a continued absence of an overarching conceptual or theoretical framework for the health humanities in health professions education, either in any single study, or emerging from what might be regarded as an international (albeit unequal) ‘community of practice’ in the area. While there was general convergence on ‘perspective’, this was largely untheorized beyond a broad notion centring on ‘empathy’. There was a persistent disconnection from critical and social studies in health and medicine being undertaken in humanities scholarship. Future developments in health humanities will benefit not only from the findings of this review but also by pushing the frontiers of what can be achieved through health humanities to address future oriented issues such as climate change and artificial intelligence.

## Supplementary Information

**Additional file 1: Supplementary Appendix**.

## Data Availability

Summarised data extracted from each individual included study is available in the supplementary material contained in Additional file 1: Appendix A. The unpublished scoping review protocol is available from the corresponding author.
