# Supplementary material for: Health Humanities curriculum and evaluation in health professions education: a scoping review
Source: BMC Med Educ. 2021 Nov 10;21:568. doi: 10.1186/s12909-021-03002-1 (PMC8579562; doi:10.1186/s12909-021-03002-1)
Supplement: Supplementary file 1 — Additional file 1: Supplementary Appendix. [file 12909_2021_3002_MOESM1_ESM.pdf]

Study Number	Authors	Title	Year of Publication	Journal	Student Population (n)	Country	Article type	Specific Discipline of Health Humanities	Study objective	Study Design	Study Analysis	Study results	Program delivery	Learning Outcome/s	Knowledge, Skills, Attitudes (KSA)	Level of Learning (Blooms)	Evaluation. Formative/ Summative	Methods	Levels of Evaluation)	Health Humanities Learning Foci
#1	Nguyen, M. et al	Arts-Based Learning: A New Approach to Nursing Education Using Andragogy.	2018	Journal of Nursing Education	Nursing (155)	Canada	Evaluation	Visual and Performing Arts	Explores the use and evaluation of andragogy-informed arts-based learning for teaching nursing theory at the undergraduate level	Mixed method	Descriptive statistics Thematic analysis	Findings suggest that arts-based learning approaches enhance learning by supporting deep inquiry and different learning styles. Further exploration of andragogy-informed arts-based learning in nursing and other disciplines is warranted.	Face to face	Skills: critical appraisal, application of theoretical approach	S	Evaluate	Formative	Post-activity questionnaires	1	2
#2	McCaffrey, G.	Bringing a novel to practice: An interpretive study of reading a novel in an undergraduate nursing practicum course.	2017	Nurse Education in Practice	Nursing (9)	Canada	Research	Literature	To elicit perspectives among participants and the interdisciplinary research team	Qualitative	Thematic analysis	Major themes that emerged included the students' tacit awareness of epistemological plurality in nursing, and the consequent importance of cultivating a capacity to move thoughtfully between different points of view and ways of knowing.	Face to face	Not documented	K	Understand	Formative	Focus Groups and interviews	2	2,3,4
#3	Thorp, L., Bassendowski, S.	Caring Values and the Simulation Environment: An Interpretive Description Study Examining Select Baccalaureate Nursing Students' Experiences.	2018	International Journal for Human Caring	Nursing (10)	Canada	Research	Performing Arts	To explore a group of nursing students' perceptions and use of the simulation environment specifically for development of caring values and abilities.	Qualitative	Iterative Thematic analysis	The results highlight that reflective practice and transformational learning transpired in these nursing students. The cocreation of knowledge in the nursing students was supported by theoretical nursing perspectives and the nursing educators. The simulation environment contributed to a safe place where nursing students could reflect and comprehend the complexity of nursing knowledge, skills, and behaviors related to caring.	Face to face	Not documented	A	Understand	Formative	Focus Groups and interviews	2	1,2,3,4
#4	Kerr AM, et al	Confessions of a Reluctant Caregiver Palliative Educational Program: Using Readers' Theater to Teach End-of-Life Communication in Undergraduate Medical Education.	2020	Health Communication	Medical (477)	USA	Evaluation	Performing arts	To examine a drama-based educational experience that focuses on the experiences of family caregivers—an often—overlooked role in end-of-life communication training.	Mixed method	Descriptive statisitics Content analysis	The results suggest the program is a valuable learning experience that is positively associated with important facets of experiential learning using narratives such as perceived realism, increased reflection, strong emotions, and increased comfort with difficult behaviors. The program offers a safe environment for medical students to identify, understand, and process the sensitive and complex issues associated with end-of-life care	Face to face	Knowledge: Understanding End of Life issues, Skills: Reflectivity	K	Understand	Not documented	Evaluation survey, cross-sectional	1, 2	1, 2
#5	Clark, Z. et al	Creating Meaningful Learning for Children's Nursing Students: Can Museum Field Trips Offer Added Value?	2019	Comprehensive Child & Adolescent Nursing	Nursing (36)	UK	Research	Visual arts	Highlights an innovative teaching strategy that was introduced to a group of year 1 children's nursing students—a field visit to the Museum of Childhood in London—and demonstrates the potential value to their learning.	Mixed method	Descriptive statisitics Content analysis	Students, stated that they felt the museum based learning linked closely with the learning outcomes of the module and the experience of seeing different displays aided their learning in a positive way. All concluded the experience was positive and the learning materials given prior to the trip were supportive in allowing students to explore the museum and gather data. The distinct but interrelated emerging themes were visual displays—deeper learning, independent working and learning, and working and learning together	Face to face	Skills:to gather information and develop research skills, communication skills	S	Create	Summative	Post-activity questionnaires	1,2	1,2,3,6
#6	Campbell BH. Et al	Creating Reflective Space for Reflective and "Unreflective" Medical Students: Exploring Seminal Moments in a Large-Group Writing Session.	2020	Academic Medicine	Medical (1,139)	USA	Research	Literature	To provide reflective and narrative-based skill-building activities for all students at the Medical College of Wisconsin (MCW)—particularly those having their initial clinical encounters	Mixed method	Correlation, Analysis of Variance, Content analysis	They misperceived their peers' views of reflective activities. Twice as many students agreed their peers felt writing, reflective, and narrative exercises were a waste of time as they themselves did (39% vs 19%). While 42% entered the session comfortable with creative writing, 57% were surprised by the amount, quality, and/or insight of their writing during the session and 77% agreed the session helped them think more clearly about clinical encounters.	Face to face	Skills: Reflection	S	Create	Formative	Post-activity questionnaires	1	2,3
#7	Greenberg RA. Et al	Developing a bioethics curriculum for medical students from divergent geo-political regions.	2016	BMC Medical Education	Medical (12)	Canada	Research	Bioethics	to determine the content and format of an ideal bioethics curriculum for a culturally diverse group of medical students.	Qualitative	Thematic analysis	While only some students had received formal ethics training prior to this program, all understood that it was a necessary and desirable subject for formal training. Interactive teaching formats were the most preferred and truth-telling was considered the most important subject.	Face to face	Knowledge- understanding bioethics, leadership, conflict resolution, global health, clinical competence and research	K	Analyse	Summative	Interview	1,2	1,2,3,4
#8	LeBlanc RG.	Digital story telling in social justice nursing education.	2017	Public Health Nursing	Nursing (108)	USA	Evaluation	Digital Narrative	to evaluate how digital stories integrated into public health nursing education can teach social justice concepts essential for nurse leadership.	Qualitative	Thematic analysis	Themes were identified based on analysis and include—Encountering Vulnerability, Questioning Systems and Choosing Moral Courage.	online	Affective:value active listening and dialog	A	Analyse	Formative	Writen reflections	2	2,3,4, 6
#9	Levin, SR. et al	Diseases, Doctors, and Divas: Cultivating Reflective Capacity in Preclinical Medical Students through a Critical Examination of Opera	2017	Journal for Learning through the Arts	Medical (13)	USA	Evaluation	Narrative	to describe the structure of the elective, the instruments used for course evaluation, and the educational outcomes in order to provide a template for using opera to develop reflective capacity and encourage use of such a paradigm at other institutions	Mixed method	Descriptive statistics Thematic analysis	The results indicate that students' awareness of how art reflects and propagates stereotypes of medicine and its practitioners was enhanced. Students reported developing an understanding of how society's perceptions of physicians have evolved by studying their representations in opera and pop culture and students appreciated learning from interdisciplinary faculty.	Face to face	Skills: Reflection	S	Analyse	Summative	Post-activity questionnaires	1,2,3	1,2,3,4
#10	Brand G. et al	Do photographs, older adults' narratives and collaborative dialogue foster anticipatory reflection ("preflection") in medical students?	2016	BMC Medical Education	Medical (128)	Australia	Evaluation	Visual and Narrative	to explore whether photographs, narratives and small group collaborative dialogue fosters reflective learning, enhances reflective capacity and has the potential to shift medical students' attitudes towards caring for older adults.	Mixed method	Descriptive, ANOVA, Thematic analysis	Quantitative and qualitative data indicated that the DOF session generated reflective learning that resulted in positive shifts in medical students' perceptions towards older adults. The qualitative reflections were captured in four main themes: the opportunity provided to Envision working with older adults; the Tension created to challenge learners' misinformed assumptions, and the work of Dismantling those assumptions, leading to Seeing older people as individuals.	Face to face	Skills: Reflection	S	Apply	Summative	Post-activity questionnaires	1,2	1, 2,3,4
#11	Komattil R. et al	Evaluation of a personal and professional development module in an undergraduate medical curriculum in India.	2016	Korean J Med Educ	Medical (528)	India	Evaluation	Ethics, Narrative, Literature	to evaluate the personal and professional development (PPD) module in the undergraduate medical curriculum in Melaka Manipal Medical College, India.	Mixed method	Descriptive statisitics Content analysis	Analysis of the course feedback form revealed that majority (80%) of students agreed that the module was well prepared and was "highly relevant" to the profession. Faculty found the topics new and interdisciplinary and there was a sense of sharing responsibility and workload by the faculty.	Face to face & online	Skills: Apply evidence synthesis and critical thinking	S	Apply	Summative	Post-activity questionnaires	1	4,5, 6
#12	Scott KM. et al	Grace Under Pressure: a drama-based approach to tackling mistreatment of medical students.	2017	Medical Humanities	Medical (30)	Australia	Evaluation	Performing Arts	to pilott three workshops developing adaptive leadership skills to help them deal with interpersonal challenges in the clinical setting, including mistreatment.	Mixed method	Descriptive statistics Thematic analysis	Workshop activities were rated as 'very good' or 'good' by 21/22 (95.5%). Thematic analysis of qualitative data highlighted the rationale for participation (to deal with bullying, prevent becoming a bully, learn social skills), workshop benefits (express emotions, learn about status dynamics and deconstructing personalities, empathy, fun), challenges (meeting participants' expectations, participants' need for further practice) and implications for medical education (need to develop awareness of others' perspectives).	Face to face	Not documented	A	Evaluate	Formative	Post-activity questionnaires, Focus Group Discussions	1	2,3,4
#13	Isaac C. et al	Impact of reflective writing assignments on dental students' views of cultural competence and diversity.	2015	Journal of Dental Eduation .	Dental (80)	USA	Research	Cultural Competence	to examine linguistic differences in dental students' relective writing assignments before and after interviewing an individual who was culturally different from themselves	Mixed method	Descriptive statistics Linguitistic Inquiry	Text analysis software identified word counts, categories, frequencies, and contexts. Signicantly positive differences occurred for interviews between assignments 1 and 2 (p=0.005 to p<0.001) in ive areas of cultural diversity. Differences were observed for Factor 1 ("important others' inluence") between assignments (p<0.001), assignments by interview categories (p=0.033), and URM/majority participants by assignments by interview category (p=0.018). Factor 4 ("my social world in relation to others") was statistically different between assignments for URM/majority participants (p=0.019). Factor 5 ("wrong because") was statistically different for gender (p=0.041), suggesting that males may have experienced a rebound effect from stereotype suppression.	Face to face	Affective:cultural competency	A	Apply	Summative	Text Analysis	2,3	1,3
#14	Saiyad SM. Et al	Introduction of Medical Humanities in MBBS 1(st) Year.	2017	Int J Appl Basic Med Res.	Medical (150)	India	Evaluation	Visual arts, Cinema series	to develop module on humanities for medical students and introduce it among 1st year MBBS students. Objectives were to sensitize and train faculty in MH, prepare a MH module, introduce MH module in 1st year MBBS, and evaluate its effectiveness.	Mixed method	Descriptive statistics Thematic analysis	According to participants' feedback and perception, mean overall rating of MH module was 4.69, indicating that it was received well by the students. Out of 3 sessions conducted, students gave maximum grades to session 2: cinemeducation. Results of faculty feedback questionnaire indicated that MH is needed and should be introduced in every batch of 1st year MBBS and should be continued longitudinally.	Face to face	Not documented	K	Understand	Not documented	Post activity questionnaires	1,2a	1,2, 3
#15	Arveklev SH. Et al	Learning About Conflict and Conflict Management Through Drama in Nursing Education.	2018	J Nurs Educ	Nursing (68)	Sweden	Research	Drama	to illuminate nursing students' experiences related to learning about conflict and conflict management through drama.	Qualitative	Content analysis	Learning about conflict and conflict management through drama enables nursing students to form new knowledge by oscillating between closeness and distance, to engage in both the fictional world and the real world at the same time. This helps students to form a personal understanding of theoretical concepts and a readiness about how to manage future conflicts.	Face to face	Skills: Communication, reflection	S	Apply	Summative	N/A	2	3
#16	McCann E, & Huntley-Moore S.	Madness in the movies: An evaluation of the use of cinema to explore mental health issues in nurse education.	2016	Nurse Educ Pract.	Nursing (22)	Ireland	Evaluation	Cinema / film	o evaluate the students' learning experience and specifically to determine the extent to which students had achieved the module learning outcomes.	Mixed method	Descriptive statistics Content analysis	All study participants agreed that their understanding of mental health issues was enriched, their attitudes and beliefs enhanced and their confidence to talk about mental health concerns increased significantly. This module provides a fruitful approach to encourage critical reflection on mental health issues in a safe environment that closely mirrors authentic practice experiences. The module facilitates the development of students' knowledge, values and attitudes in relation to person-centred mental healthcare.	Face to face	Affective: value communication, Teamwork	A	Understand	Summative	Text Analysis	1, 2a, 2b	1,2,3
#17	Patterson A. et al	Medical humanities: a closer look at learning.	2016	Med Humanities	Medical (156)	UK	Research	Reflective writing	to investigate what students learned from participating in a short medical humanities student-selected module in their first year of an undergraduate medical programme.	Mixed method	Descriptive statistics Content analysis	The reflection content analysis supported the heterogeneous nature of learning outcome for students, with evidence to support the idea that the module provided opportunities for students to explore their beliefs, ideas and feelings regarding a range of areas outside their current experience or world view, to consider the views of others that they may have not previously been aware of, to reflect on their current views, and to consider their future professional practice.	Face to face	Not documented	K	Understand	Summative	N/A	2a	2
#18	Centeno, C. et al	Palliative care and the arts: vehicles to introduce medical students to patient-centred decision-making and the art of caring.	2017	BMC Med Education	Medical (20)	Spain	Evaluation	Visual Arts	to describe the curriculum and evaluation results of a unique course centred on palliative care decision-making but aimed at introducing these other important competencies as well	Mixed method	Descriptive statistics Thematic analysis	Students confirmed the relevance of the course content (mean 5/5) and the course was rated very positively across a number of parameters. The course also received high scores relative to other courses that year.	Face to face	Affective:open mindedness; self-awareness and appreciating the different perspectives	A	Understand	Summative	Post-activity questionnaires, Focus Group Discussions	1,2a,2b	1,2,3
#19	Rice, R. et al	Perceptions of Nursing Students Regarding Usage of Art Therapy in Mental Health.	2017	Journal of Nursing Education	Nursing (32)	USA	Research	Visual Arts	to describe the perceptions of undergraduate nursing students regarding the use of art therapy on the therapeutic relationship and communication between nursing students and mental health patients?	Qualitative	Thematic analysis	Major themes found in the study included: Nursing Students' Initial Experiences With Mental Health Patients, Nursing Students' Observations of Mental Health Patients, and Nursing Students' and Mental Health Patients' Responses to Art Therapy.	Face to face	Not documented	K	Apply	Summative	N/A	1	3
#20	Thompson,BM. Et al	Providing context for a medical school basic science curriculum: The importance of the humanities.	2015	Medical Teacher	Medical (132)	USA	Research	Visual Arts / Performing Arts / Literature/Philosophy	to gain insight into how first- and second-year medical students' views of being a doctor evolved after participating in a required eight week humanities course.	Qualitative	Thematic analysis	A majority of first- and second-year medical students (rr¼70%) replied, resulting in 100 pages of text. A meta-theme of Contextualizing the Purpose of Medicine and three subthemes: the importance of Treating Patients Rather than a Disease, Understanding Observation Skills are Important, and Recognizing that Doctors are Fallible emerged from the data.	Face to face	Skill: Reflective Writing	K	Understand	Summative	N/A	1,2a	1,2,3,4
#21	Gilkison,A. et al	Real life narratives enhance learning about the 'art and science' of midwifery practice.	2015	Advances in Health Sciences Education	Midwifery (14)	New Zealand	Research	Narrative	to answer ''what is the experience of midwifery teachers and students who participated in a narrative-centred curriculum?''	Qualitative	Narrative Interpretation	This study found that when narratives were central to the learning environment, students reflected on knowledge, values and beliefs, and related those to the narrative and midwifery practice; a skill which is the cornerstone of phronesis (the art of practice).Students' focus groups and written reflections showed that they learned about themselves, others, and midwifery practice, through experiencing emotional responses which were aroused by the narratives.	Face to face	Attitudes: value perspectives, know their own values	A	Understand	Summative	N/A	1,2a,b,3	2,3,4
#22	Emmanuel EN.	Teaching Humanity to Nursing Students: Evaluation of an Innovative Teaching Strategy.	2016	Nurse Educ. 2016	Nursing (143)	Australia	Evaluation	Visual arts	to explain how an art-based teaching assessment was used in an undergraduate nursing course on mental health.	Qualitative	Thematic analysis	Results showed predominantly higher-order thinking at multistructural level and higher. Essentially, this suggested that students were able to demonstrate strong levels of analysis, evaluation, synthesis, and creation.	Face to face	Skills: observation, communication,teamwork	A	Understand	Summative	Biggs SOLO taxonomy text analysis	2a, 2b	1,2,3,4,6
#23	Day L. et al	The power of nursing: An innovative course in values clarification and self-discovery.	2017	J Prof Nurs	Nursing (68)	USA	Evaluation	Visual arts	to describe the development, implementation and evaluation of a learning innovation for pre-RN students.	Mixed method	Descriptive statistics Thematic analysis	Most agreed the course provided educational experiences not available elsewhere in curricula (4.74; SD 0.52), all disagreed the small group experiences were similar to others in the program (1.79; SD 0.85), all agreed each session was important to their nursing practice development (4.6-4.77; SD 0.47-0.78).	Face to face	Not documented	A	Apply	Summative	Post-activity questionnaires	1,	2,3,4
#24	Haidet P. et al	Using Jazz as a Metaphor to Teach Improvisational Communication Skills.	2017	Healthcare	Medical (34	USA	Evaluation	Music	to investigate the effects of using jazz as a metaphor to teach senior medical students improvisational communication skills, and to understand student learning experiences	Mixed method	Descriptive statistics Thematic analysis	Student self-assessments of knowledge improved on all four global knowledge items (knowledge of jazz., enjoymentof jazz, undestanding of improvisation, understanding of pt-physcian communication).Student self-assessments improved on a composite rating of seven abilities related to the objectives of the course. Student attitudes toward patient-centered care and mindful practice did not change over the period of the course. Students' ratings of confidence in completing essential communication tasks improved over the period of the course. Standardised pt putcomes: The course group demonstrated significant gains from pre- to post-course in both adaptability and quality of listening. While there were no significant differences between the course and control groups on the pre-course measures, the course group scored higher on the post-course adaptability evaluation, and gained significantly more than the control group on the listening evaluation. Cohen's D scores indicated large effect sizes (d > 0.8) for all statistically [illegible]	Face to face	Skills: Communication, reflection	S	Apply	Summative	Post-activity questionnaires, Interviews	1, 2a, 2b, 4	1,2,3,
